# Route-Dependent Mucosal and Systemic Immune Remodeling Induced by a Regulated-Lysis *Edwardsiella piscicida* Vaccine in Channel Catfish

**DOI:** 10.3390/vaccines14050410

**Published:** 2026-05-01

**Authors:** Kavi R. Miryala, Roy Curtiss, Vinicius Lima, Banikalyan Swain

**Affiliations:** Department of Infectious Diseases & Immunology, College of Veterinary Medicine, University of Florida, Gainesville, FL 32608, USA; kavi.miryala@ufl.edu (K.R.M.);

**Keywords:** *Edwardsiella piscicida*, RAEV, channel catfish, regulated-lysis vaccine, immersion vaccination, mucosal immunity, transcriptomics, aquaculture, intestinal immune response

## Abstract

**Background:** *Edwardsiella piscicida* is a significant intracellular pathogen of channel catfish (*Ictalurus punctatus*) and a major threat to U.S. aquaculture. A recently developed recombinant attenuated vaccine strain (χ16016) uses arabinose-regulated *murA* expression to trigger delayed cell wall lysis in vivo, ensuring biological containment while conferring strong protection against virulent challenge. Although its efficacy has been demonstrated, the host immune programs underlying protection remain incompletely defined. **Methods:** We used RNA sequencing to characterize tissue-specific transcriptomic responses in the intestines and kidneys of channel catfish at 7 days post-vaccination. Fish were vaccinated with χ16016 by either bath immersion or intracoelomic (IC) injection, and differentially expressed genes and enriched immune pathways were analyzed to determine how the vaccine delivery route shapes systemic and mucosal immune responses. **Results:** Across comparisons, 19,101 differentially expressed genes revealed pronounced route- and tissue-dependent immune remodeling. As aquaculture vaccination strategies increasingly prioritize scalability and practical deployment, understanding how the delivery route shapes immune outcomes is critical. Here, IC vaccination induced broader systemic transcriptional changes, particularly in the intestine, whereas bath immunization elicited a more focused yet coordinated mucosal response. Overall, intestinal tissue exhibited greater transcriptional responsiveness than kidney tissue, underscoring its central role in early vaccine-induced immunity. Functional enrichment analyses identified the activation of innate recognition pathways, MAPK and calcium signaling cascades, complement components, antigen processing machinery, and cell adhesion networks. Notably, bath immunization enriched the *intestinal immune network for IgA production* pathway, which represents an orthology-based mapping of conserved mucosal immune components, alongside the upregulation of IL-6, CXCL12–CXCR4, integrins (α4β7), MHC class II, complement C3, and polymeric immunoglobulin receptor (pIgR). Given that catfish rely primarily on IgM in mucosal immunity, these findings indicate the induction of IgM-mediated mucosal defense rather than classical mammalian IgA responses. Concurrent complement and scavenger receptor signatures suggest a transition toward efficient opsonophagocytic clearance with controlled inflammation at this subacute stage. **Conclusions:** This study provides the first systems-level view of host transcriptomic responses to a regulated-lysis *E. piscicida* vaccine in channel catfish. The findings demonstrate that immersion vaccination, although transcriptionally less expansive than injection, effectively activates coordinated mucosal innate and adaptive immune programs, supporting its practical use as a scalable vaccination strategy for aquaculture.

## 1. Introduction

*Edwardsiella piscicida* is an emerging Gram-negative facultative intracellular pathogen, responsible for edwardsiellosis, a septicemic disease impacting a variety of warm-water teleost fish, including channel catfish (*Ictalurus punctatus*) [1,2]. Outbreaks of *E. piscicida* in aquaculture lead to high fry and fingerling mortality and substantial economic losses in the U.S. catfish industry, with *Edwardsiella* spp. infections resulting in up to USD 24.8 million in lost revenue annually [3]. The overuse of antibiotics to control such infections has raised concerns about drug resistance and environmental impacts, making prophylactic measures such as vaccination a sustainable method of eliciting immune protection across a variety of pathogens in aquaculture [4].

Live attenuated bacterial vaccines have generally been shown to elicit more reliable and long-lasting protective immunity while limiting the need for boosters when compared to their inactivated and subunit counterparts; they act by mimicking natural infections [1]. Among these, recombinant live attenuated *E. piscicida* vaccines (RAEVs) have shown promise in protecting fish against edwardsiellosis [2,5].

A central challenge in live vaccine development is achieving a balance between attenuation and immunogenicity. Traditional gene knockout approaches can over-attenuate the pathogen, limiting its ability to colonize host tissues and stimulate immunity [6]. To address this, several studies have pioneered regulated delayed attenuation strategies for *E. piscicida* vaccines, adapting genetic frameworks originally developed for *Salmonella* spp. and *Edwardsiella* spp. vaccine platforms [2,5,7,8,9,10,11,12]. In this approach, an essential gene is placed under the control of an inducible promoter, allowing the vaccine strain to replicate transiently in the presence of an exogenous stimulus but forcing its attenuation in vivo when the stimulus is absent (i.e., within the host) [12].

The development of these vaccines has proceeded through sequential modifications of this attenuation strategy across different genetic targets to improve control over attenuation while maintaining immunogenicity. For example, placing the global regulator gene *fur* under an arabinose-dependent promoter yielded an *E. piscicida* strain that behaves like the wild type during initial immunization but then ceases *fur* expression once inside the host, leading to the gradual loss of virulence and prevention of disease [12]. This nutrient-regulated attenuation framework has been extended to additional targets by Swain et al. (2020 and 2022a), including metabolic and virulence-associated genes such as *aroA* and *phoP*, as well as systems involving global regulators such as *crp*, demonstrating the flexibility of this approach across vaccine designs [12]. Building on this framework, Swain et al. (2022b) developed a multifaceted RAEV vector strain of *E. piscicida*, χ16022, incorporating multiple safety features, including the deletion of *asdA* and arabinose-regulated expression of *fur* and *crp*, and further engineered it to express a heterologous antigen from *Ichthyophthirius multifiliis*, creating a bivalent vaccine platform [5]. This strain demonstrated the controlled attenuation and induction of both systemic and mucosal immune responses, resulting in approximately 60% survival following challenge, and helped to establish a generalizable framework in which regulated attenuation strategies can confer safety without compromising immunogenicity [5].

Most recently, Swain et al. (2023) developed the χ16016 strain, which serves as the basis for the present study. It was derived from the wild-type *E. piscicida* strain J118, a highly virulent fish isolate derived from EIB202, which was previously sequenced and characterized [2,13]. χ16016 was developed from the J118 strain by engineering a deletion–insertion mutation in the *murA* promoter region. Briefly, a 595 bp upstream fragment of *murA* and a 499 bp downstream fragment carrying a modified Shine–Dalgarno (SD) sequence were amplified from J118 genomic DNA, assembled with the *araC* P_araBAD_ cassette in the suicide vector pRE112, and introduced into J118 by conjugation from *Escherichia coli* χ7213 [2]. Mutants were selected by sacB-based sucrose counterselection and confirmed by PCR and sequencing [2]. This replaced the native *murA* promoter with the tightly regulated araC ParaBAD cassette, placing the expression of the essential cell wall biosynthesis gene *murA* under arabinose-dependent control. In arabinose-supplemented medium, χ16016 grows normally because *murA* is expressed and peptidoglycan synthesis is maintained [2]. After entry into fish tissues, where arabinose is unavailable, *murA* expression is shut off, peptidoglycan synthesis cannot continue, and the bacteria undergo progressive lysis in vivo [2]. The design enables transient colonization and antigen delivery while providing biological containment by preventing the long-term persistence of the vaccine strain.

This conditional lethality showed significantly higher survival in immunized catfish, with relative percent survival ~80% compared to ~30% in unvaccinated controls upon virulent *E. piscicida* (wild-type J118 strain) challenge [2]. Immune profiling in the study revealed that the vaccine elicited strong innate and adaptive responses: vaccinated catfish had upregulated pattern recognition receptors (TLR4, TLR5, TLR8, TLR9, NOD1, NOD2) that activated NF-κB pathways, along with proinflammatory cytokines (*tnf-α*, *il-1β*, *il-6*, *ifn-γ*) and Th-cell markers (*cd4*, *cd8*, *mhc-II*) in lymphoid organs [2]. The induction of systemic and mucosal IgM responses further underscores the immunogenicity of this platform, establishing the murA-regulated χ16016 strain as a central model for investigating host immune mechanisms in this study [2].

Despite these advances, the in vivo immune mechanisms conferring protection remain only partially understood. The previous work by Swain et al. (2023) relied on targeted gene expression assays (e.g., qPCR for select cytokines and receptors), which do not capture the full breadth of the host’s response to vaccination [2]. In particular, little is known about the global transcriptomic changes induced by live attenuated vaccines in catfish or how different vaccination routes may influence the immune landscape in various tissues.

Challenge protection was not re-evaluated in the present study because the safety, immunogenicity, and protective efficacy of χ16016 in channel catfish have already been established, including a significant survival advantage following virulent *E. piscicida* challenge. Instead, the present study was designed to characterize early tissue-specific transcriptomic responses following vaccination, providing a foundation for future mechanistic studies.

In this study, our objective was to define the host immune programs engaged by a *murA*-regulated live attenuated *E. piscicida* vaccine at an early post-vaccination stage, building upon the foundations of Swain et al. (2023) and earlier works [2]. Using transcriptomic analysis, we aimed to identify molecular pathways and regulatory networks that are associated with vaccination and could inform future mechanistic studies of this regulated-lysis vaccine platform in channel catfish.

Here, we employed RNA sequencing (RNA-seq) to comprehensively profile host gene expression in vaccinated versus unvaccinated channel catfish. We therefore focused on two immunologically distinct and complementary tissues: the kidney, which is the primary hematopoietic and systemic immune organ in teleosts, and the intestine, as a major mucosal immune interface. We compared two vaccination routes that are both widely used and operationally relevant in aquaculture, namely intracoelomic injection and bath immersion, which differ fundamentally in antigen exposure, uptake, and systemic dissemination. It should be noted that this study focused on post-vaccination immune responses rather than post-challenge efficacy, and sampling was limited to a single time point at 7 days post-vaccination (dpv), as the previous study by Swain et al. (2023) had already characterized immune kinetics at 3, 5, and 7 days post-vaccination, establishing the temporal profile of early innate and adaptive activation [2]. These observations contribute to the growing understanding of how attenuated vaccines interact with host immunity in teleost fish and can guide the refinement of recombinant live attenuated vaccine strategies for aquaculture.

## 2. Materials and Methods

### 2.1. Culture and Preparation of Edwardsiella piscicida RAEV Strain χ16016 for Vaccination

The RAEV strain χ16016, previously derived from wild-type *Edwardsiella piscicida* strain J118, harbors a deletion–insertion mutation in the *murA* promoter region (ΔP_murA180_::TT *araC* P_araBAD_ *murA*) [5]. The strain was initially streaked onto LB agar plates supplemented with 0.2% L-arabinose and incubated overnight at 30 °C. A single colony was then used to inoculate 2 mL of LB broth containing 0.2% L-arabinose, and the culture was incubated at 30 °C with shaking at 180 rpm overnight. Subsequently, 1 mL of the overnight culture was transferred into 100 mL of fresh LB broth supplemented with 0.2% L-arabinose and incubated under the same conditions (30 °C, 180 rpm) until the culture reached the log phase. Following incubation, the culture was adjusted to a concentration of 5 × 10^4^ CFU/100 µL in BSG for intracoelomic (IC) injection and to 5 × 10^6^ CFU/mL in tank water for bath immersion exposure. It should be noted that bath and intracoelomic vaccination differ not only in route but also in the antigen dose, duration of exposure, handling intensity, and use of anesthetic sedation; however, these procedural differences are inseparable features of each delivery method as they are practiced in commercial aquaculture and represent the two most operationally relevant immunization strategies in catfish farming. As such, these inherent differences preclude the attribution of transcriptional divergence exclusively to the route of antigen entry, and comparisons between delivery methods should be interpreted within this practical context.

### 2.2. Fish Husbandry

All animal procedures were approved by the Institutional Animal Care and Use Committee (IACUC) at the University of Florida (Protocol #IACUC202300000342). Clinically healthy channel catfish (*Ictalurus punctatus*) fingerlings (2.5 ± 0.5 g) were obtained from Osage Catfisheries (Osage Beach, MO, USA) and acclimated in the laboratory for 2 weeks. Fish were maintained in 40 L tanks at a density of 35 fish per tank and held at 26 ± 2 °C. Reverse osmosis (RO) water was conditioned with Instant Ocean^®^ sea salt (Instant Ocean, Blacksburg, VA, USA) and sodium bicarbonate to achieve conductivity of 300–400 µS and a pH of 7.0–7.4. A commercial pelleted diet was provided twice daily, and a 14 h light–10 h dark photoperiod was maintained to simulate natural environmental cues.

### 2.3. Immunization

Channel catfish fingerlings were assigned to four treatment groups: (1) bath control, (2) bath immunized with χ16016, (3) IC control (vehicle only), and (4) intracoelomic (IC) immunized with χ16016. Each group was maintained in triplicate tanks, with 15 fish per tank (*n* = 45 per group), under the same environmental conditions. The sample size was based on prior studies and standard practices in similar transcriptomic experiments, and no animals or samples were excluded from analysis.

The bacterial strain used for immunization, χ16016, is a lysis-attenuated mutant previously shown to be immunogenic in catfish [5]. Before IC vaccination, fish were sedated with SYNCAINE^®^ (MS-222) (Syndel, Ferndale, WA, USA) at a concentration of 40 mg/L to minimize handling stress. Fish were then injected intracoelomically with 5 × 10^4^ CFU of χ16016 in a 100 µL volume, using a ventral injection site posterior to the pelvic fins. Bath immunization was performed by immersing fish in an aerated tank containing 5 × 10^6^ CFU/mL of χ16016 for 2 h at 26 °C. Control fish received either a sterile buffer (BSG) through IC injection or immersion in water, mirroring the conditions of their respective treatment groups. At 7 days post-vaccination, kidney and intestinal tissues were collected from fish. Samples were immediately preserved in RNAlater^®^ and stored at −80 °C for transcriptomic analysis.

### 2.4. RNA Isolation

Total RNA was isolated from kidney and intestinal tissues using TRIzol™ Reagent (Invitrogen, Carlsbad, CA, USA), following the manufacturer’s instructions. After dissection, tissues were preserved in RNAlater^®^ (Thermo Fisher Scientific, Waltham, MA, USA) at −80 °C until processing. For each group, tissues from three individual fish were pooled to create one biological replicate. RNA quality and concentration were assessed using a NanoDrop™ spectrophotometer (Thermo Fisher Scientific, Waltham, MA, USA), and integrity was confirmed by agarose gel electrophoresis based on the presence of intact 28S and 18S rRNA bands. Samples were treated with 1 U of DNase I (Thermo Fisher Scientific, Waltham, MA, USA) to remove residual genomic DNA. All steps were performed using RNase-free reagents and equipment. Purified RNA was stored at −80 °C until transcriptomic analysis.

### 2.5. Transcriptome Analysis

Total RNA samples were submitted to Novogene (Beijing, China) for transcriptome sequencing. Differential expression analyses included the following six comparisons: (1) bath-immunized kidney vs. bath control kidney (Bath K vs. Bath CK), (2) bath-immunized intestine vs. bath control intestine (Bath I vs. Bath CI), (3) intracoelomic-immunized kidney vs. intracoelomic control kidney (IC K vs. IC CK), (4) intracoelomic-immunized intestine vs. intracoelomic control intestine (IC I vs. IC CI), (5) bath-immunized intestine vs. intracoelomic-immunized intestine (Bath I vs. IC I), and (6) bath-immunized kidney vs. intracoelomic-immunized kidney (Bath K vs. IC K).

**Figure 1:**
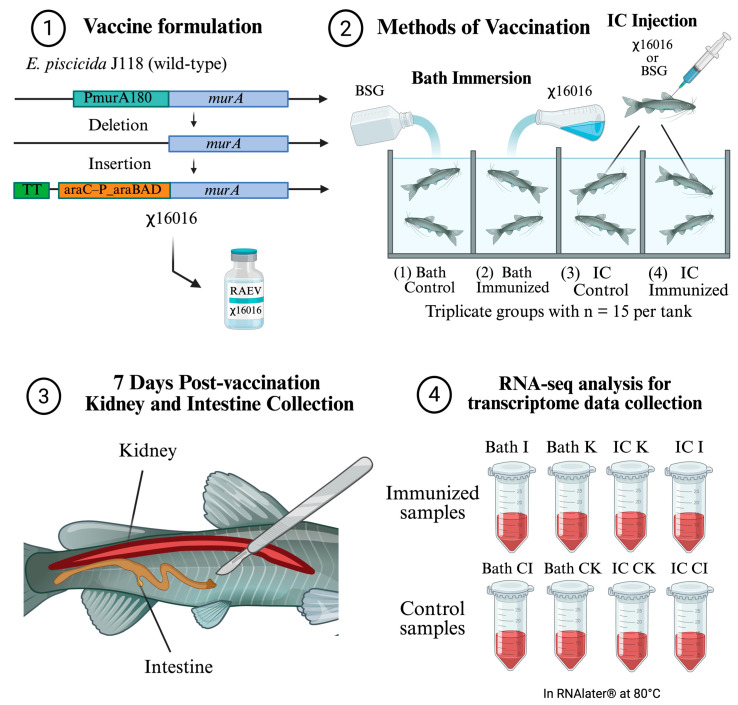
This schematic outlines the study design used to evaluate host responses to the recombinant attenuated *E. piscicida* vaccine (RAEV) strain χ16016. (1) The attenuated vaccine strain χ16016 was derived from wild-type *E. piscicida* J118 using a targeted deletion–insertion genetic modification (ΔP_murA180_::TT *araC* P_araBAD_ *murA*). (2) Channel catfish were immunized either by bath immersion (Bath) with χ16016 or BSG as a control or by IC injection using the same treatments, which can be referenced in Section 2.3. (3) To examine tissue-level responses to vaccination, kidney and intestine samples were collected at 7 days post-vaccination (dpv). (4) RNA sequencing of these samples produced eight transcriptomic datasets corresponding to Bath I (bath-immunized intestine), Bath K (bath-immunized kidney), IC I (IC-immunized intestine), IC K (IC-immunized kidney), Bath CI (bath-immunized control intestine), Bath CK (bath-immunized control kidney), IC CI (IC-immunized control intestine), and IC CK (IC-immunized control kidney). Although eight transcriptomic datasets were generated, differential expression analysis was conducted using six biologically relevant comparison groups derived from these samples (CI, CK, I, and K across bath and intracoelomic routes). We used this framework to compare tissue-specific immune responses across vaccination routes after χ16016 vaccination, using transcriptomic data associated with vaccine-mediated protection. Figure created with BioRender.com.

For each treatment group, three independent pooled biological replicates were prepared for each tissue, with each pool consisting of tissue from three fish. Each pooled replicate was used to generate one RNA-seq library for sequencing. Libraries were prepared using poly(A) enrichment, strand-specific cDNA synthesis, and the ligation of Illumina-compatible adapters. Sequencing was performed on the Illumina NovaSeq (Illumina, San Diego, CA, USA) 6000 platform, generating 150 bp paired-end reads. Raw reads were filtered to remove adapter sequences, low-quality reads, and reads with ambiguous bases. Clean reads were aligned to the *Ictalurus punctatus* reference genome (NCBI RefSeq assembly GCF_001660625.3) using HISAT2 (v2.2.1). Gene-level quantification was performed using featureCounts (v2.0.8), and differential expression analysis was carried out with DESeq2 (v1.46.0) in R (v4.4.3), using |log_2_ fold change| ≥ 1 and adjusted *p*-value < 0.05 (Benjamini–Hochberg correction) as significance thresholds. Gene Ontology (GO) and Kyoto Encyclopedia of Genes and Genomes (KEGG) enrichment analyses were conducted using the clusterProfiler package (v4.14.6) in R (v4.4.3). Results were visualized with volcano plots and heatmaps to highlight differentially expressed genes and enriched biological pathways. The simplified overall methodology can be found in Figure 1.

## 3. Results

### 3.1. Comparative Analysis of Tissue-Specific Transcriptomic Responses to Bath and Intracoelomic Immunization in Channel Catfish

**Figure 2:**
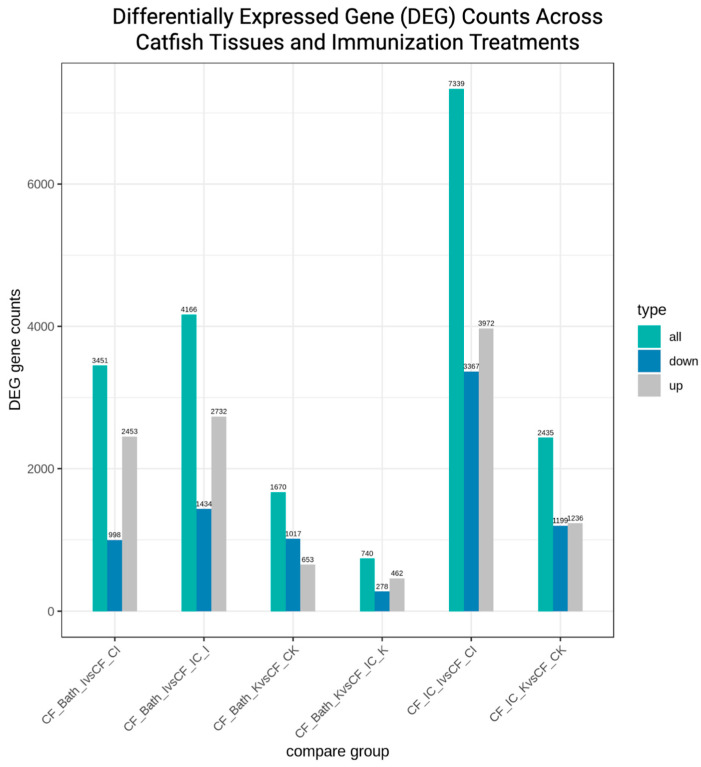
Differentially expressed gene (DEG) counts in channel catfish following bath and intracoelomic immunization. The bar graph shows the total number of differentially expressed genes (DEGs) identified in the intestinal and kidney tissues of channel catfish after bath and intracoelomic (IC) immunization compared with unvaccinated controls. Comparison groups are defined as control intestine (CI), control kidney (CK), immunized intestine (I), and immunized kidney (K). Teal bars represent total DEGs, blue bars indicate downregulated genes, and gray bars indicate upregulated genes.

Following differentially expressed gene (DEG) analysis, a total of 19,101 DEGs were identified across the six tissue and treatment comparisons (Figure 2). Differential gene expression analysis revealed clear route- and tissue-specific transcriptomic responses in channel catfish following bath and intracoelomic (IC) immunization. Overall, IC immunization induced a markedly stronger transcriptional response than bath immunization, particularly in the intestinal tissue.

The most pronounced response was observed in the intestine following IC immunization (IC_I vs. IC_CI), with 733

9 differentially expressed genes (DEGs), comprising 3972 upregulated and 3367 downregulated genes. This relatively balanced distribution suggests the coordinated activation and regulation of immune-related pathways.

Bath immunization elicited more moderate transcriptional changes. In the intestine, 3451 DEGs (2453 upregulated; 998 downregulated) were identified when compared with the control intestine (Bath_I vs. Bath_CI), while 4166 DEGs (2732 upregulated; 1434 downregulated) were observed when the bath-immunized intestine was compared with the IC-immunized intestine (Bath_I vs. IC_I). In the kidney, bath immunization resulted in 1670 DEGs (653 upregulated; 1017 downregulated) relative to the control kidney (Bath_K vs. Bath_CK). Meanwhile, bath immunization, when compared with the IC-immunized kidney (Bath_K vs. IC_K), displayed 740 DEGs (462 upregulated; 278 downregulated). This lower number likely indicates the differential expression of similar genes between the two immunization routes in the kidney.

Moreover, these results indicate that kidney tissue consistently exhibited fewer DEGs than the intestine across both immunization routes, as the IC immunization of the kidney (IC_K vs. IC_CK) showed 2435 DEGs (1236 upregulated; 1199 downregulated), indicating a substantial but less extensive response than that observed in the intestine. Across bath immunization comparisons, upregulated genes generally outnumbered downregulated genes, whereas IC immunization produced a more balanced pattern of up- and downregulation. Collectively, these findings demonstrate that IC delivery, along with its associated procedural differences, affects a larger number of genes with both activating and repressive changes compared to bath exposure, with the intestine showing greater transcriptional sensitivity than the kidney.

Intracoelomic immunization induced the strongest transcriptional response, particularly in the intestine (I vs. CI). Bath immunization produced more moderate gene expression changes across tissues. Overall, kidney samples exhibited fewer DEGs than intestinal samples under both immunization routes. The relative proportions of upregulated and downregulated genes highlight clear route-dependent and tissue-specific immune responses.

### 3.2. Route-Dependent and Tissue-Specific Transcriptomic Divergence Following Bath and Intracoelomic Immunization in Channel Catfish

**Figure 3:**
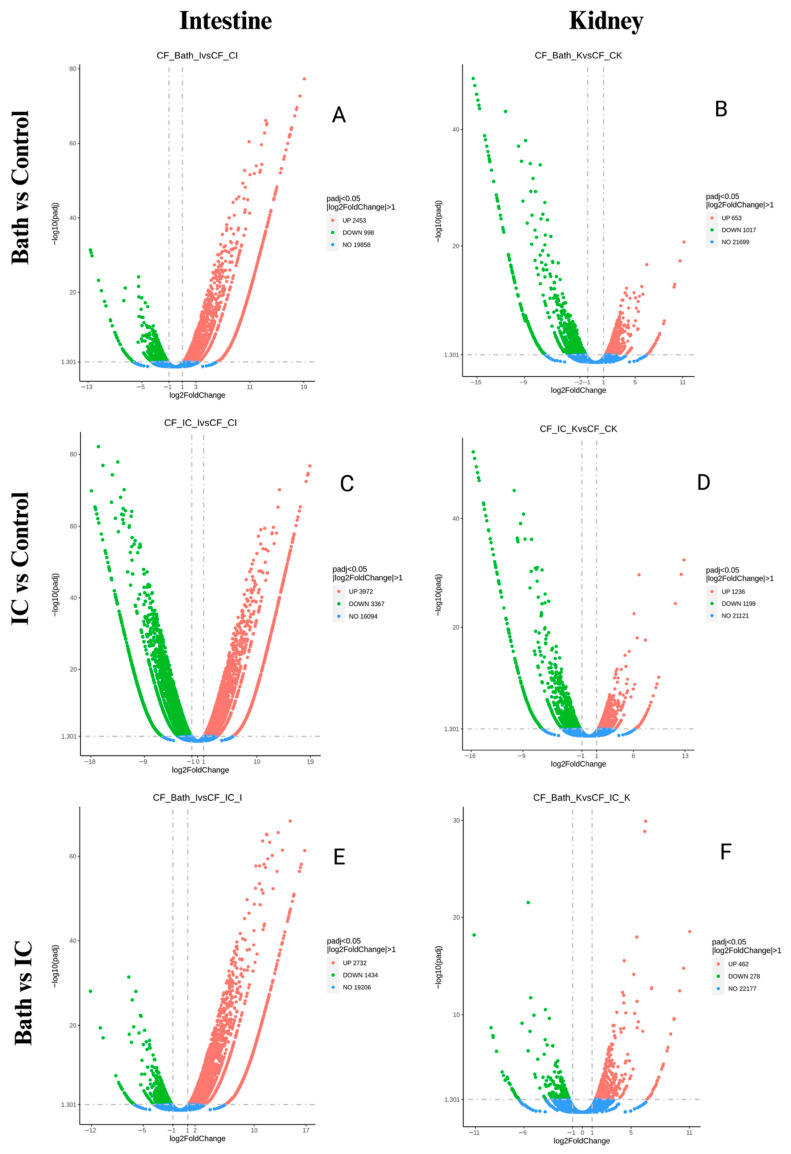
Volcano plots showing differential gene expression in intestinal and kidney tissues following bath and intracoelomic immunization in channel catfish. Volcano plots illustrate differentially expressed genes (DEGs) identified after bath and intracoelomic (IC) immunization. Genes were considered significant at an adjusted *p*-value (padj) < 0.05 and |log_2_ fold change| > 1, as indicated by the horizontal and vertical dashed threshold lines, respectively. Each point represents a single gene plotted by the log_2_ fold change (x-axis) against −log_10_(padj) (y-axis). Upregulated genes are shown in red, downregulated genes in green, and non-significant genes in blue. Panels (**A**,**B**) represent bath versus control comparisons for the intestine and kidney, respectively. Panels (**C**,**D**) show IC versus control comparisons, and Panels (**E**,**F**) present direct comparisons between bath and IC immunization in intestine and kidney tissues. Intestinal samples (**A**,**C**,**E**) display broader dispersion and a greater number of significantly regulated genes than kidney samples (**B**,**D**,**F**), indicating stronger mucosal transcriptional responsiveness. Overall, the plots highlight clear route-dependent (from a practical, commercial delivery perspective) and tissue-specific differences in vaccine-induced gene expression.

Volcano plot analysis was used to visualize the magnitude and direction of transcriptional changes induced by bath and IC immunization in intestinal and kidney tissues (Figure 3). Across all comparisons, significantly upregulated and downregulated genes were clearly separated (Panels A–F), supporting the differential expression patterns identified in the DEG analysis.

In intestinal tissue, IC immunization produced a wide distribution of both upregulated and downregulated genes (Panel C), indicating coordinated activation and repression across multiple transcriptional programs, whereas bath immunization showed prominent upregulation with comparatively fewer strongly downregulated transcripts (Panel A). In contrast, kidney tissue exhibited a narrower range of fold-change values under both immunization routes (Panels B and D), consistent with a more constrained transcriptional response.

Direct comparisons between IC and bath immunization further demonstrated pronounced route-dependent divergence in the intestine (Panel E), while kidney profiles displayed fewer genes with large fold changes (Panel F). However, this broader transcriptional dispersion following IC immunization may partly reflect systemic exposure and injection-associated stress rather than route-specific immune activation alone. Overall, volcano plot patterns revealed clear tissue-specific differences in transcriptional regulation, showing greater fold-change dispersion and likely route-dependent divergence in intestinal tissue compared with the more constrained responses observed in kidney tissue.

### 3.3. Gene Ontology (GO) Enrichment Analysis

To characterize the functional implications of transcriptional changes, GO enrichment analysis was performed using differentially expressed genes (DEGs) from each comparison. The ten most significantly enriched terms from each GO category—Biological Process (BP), Cellular Component (CC), and Molecular Function (MF)—were selected, yielding 30 representative terms per comparison (Supplementary Figure S1).

#### 3.3.1. GO Analysis in Intestinal Tissue

In intestinal tissue, bath immunization primarily enriched terms associated with innate defense and tissue protection, including response to drug (27 DEGs), blood coagulation (22 DEGs), and hemostasis (23 DEGs). These categories suggest the activation of early inflammatory and barrier-protective mechanisms.

Intracoelomic (IC) immunization, in contrast, showed strong enrichment in structural and epithelial integrity-related categories, such as cell adhesion (181 DEGs), cell junction (143 DEGs), and bicellular tight junction (47 DEGs). These enrichments indicate enhanced mucosal barrier remodeling and coordinated epithelial immune signaling following IC administration.

Direct comparison of the immunization routes (Bath vs. IC) revealed enrichment largely dominated by muscle contraction-related processes, suggesting that many of the observed differences between delivery methods reflect broader physiological modulation, including potential stress responses associated with handling and sedation, rather than classical immune pathway activation alone. Nonetheless, immune-associated terms such as receptor ligand activity (82 DEGs) were also enriched, indicating subtle but meaningful differences in mucosal immune recognition and signaling between delivery methods.

Overall, intestinal GO patterns demonstrate robust mucosal responsiveness, particularly following IC immunization.

#### 3.3.2. GO Analysis in Kidney Tissue

In kidney tissue, both bath and IC immunization relative to controls produced enrichment patterns dominated by structural and reproductive-associated categories, reflecting broader physiological modulation rather than strong immune activation.

Bath immunization uniquely enriched receptor ligand activity (38 DEGs), suggesting the modest modulation of renal immune signaling pathways.

Notably, the direct comparison between bath and IC immunization in kidney tissue displayed the greatest diversity of immune-related GO categories. Enriched terms included immune response (20 DEGs), defense response (12 DEGs), response to bacterium (7 DEGs), response to oxidative stress (5 DEGs), cytokine/chemokine activity (12 DEGs), and cytokine/chemokine receptor binding (11 DEGs). However, despite broader immune category representation, the DEG counts within these terms were relatively modest, indicating limited but distinct route-dependent immune modulation in the kidney.

Importantly, gene regulation patterns (Supplementary Figure S2) revealed that most immune-related DEGs in this comparison were upregulated, suggesting that bath immunization tends to promote higher renal immune gene expression relative to IC vaccination.

Collectively, the kidney GO results indicate more restrained transcriptional remodeling compared to the intestine, with subtle yet route-specific immune modulation.

### 3.4. KEGG Pathway Enrichment Analysis

To further contextualize the functional significance of these transcriptional changes, Kyoto Encyclopedia of Genes and Genomes (KEGG) pathway enrichment analysis was performed. This analysis identified major immune and signaling pathways associated with vaccination, providing a functional context for the molecular processes associated with route- and tissue-specific immune responses.

**Figure 4:**
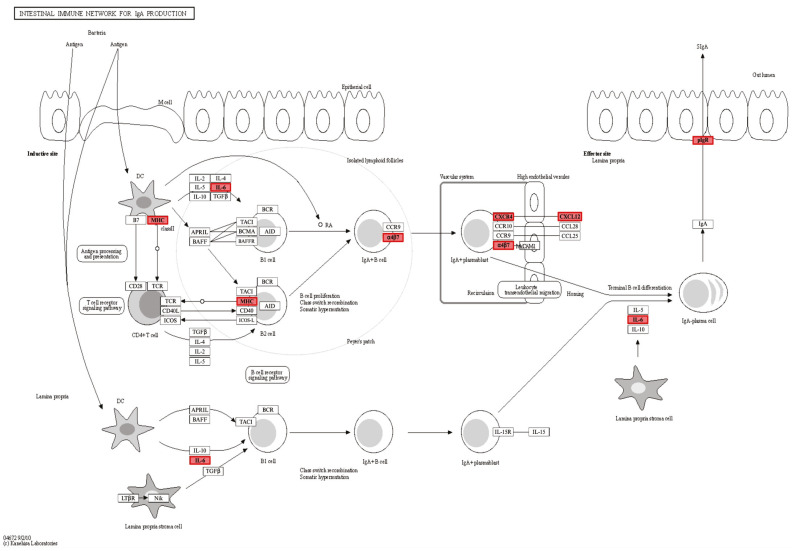
KEGG pathway map of the *intestinal immune network for IgA production* in bath-immunized channel catfish. Schematic representation of the Kyoto Encyclopedia of Genes and Genomes (KEGG) pathway for the intestinal immune network involved in immunoglobulin A (IgA) production, representing a KEGG-defined pathway used here as a proxy for mucosal antibody signaling in catfish, likely representing IgM. The pathway highlights differentially expressed genes (DEGs) identified in the bath intestine versus control intestine comparison (Bath_I vs. Bath_CI) at 7 days post-vaccination (dpv). Red-colored genes indicate upregulation, and no downregulation was identified in this figure. Arrows indicate the direction of signaling or cellular movement within the pathway, and gene names positioned along arrows represent mediators of the indicated interaction or process. All other relevant components, including cell types, are labeled.

To provide a functional context for transcriptional changes induced by vaccination, a KEGG pathway enrichment analysis of the *intestinal immune network for IgA production* pathway (orthologous KEGG pathway reflecting conserved mucosal immunoglobulin mechanisms, likely IgM-mediated in catfish) was performed using the bath intestine versus control intestine comparison (Bath_I vs. Bath_CI) as a representative dataset (Figure 4). This comparison was selected because bath immunization represents a non-invasive, scalable, and industry-relevant vaccination strategy in aquaculture. Moreover, the intestine serves as the primary mucosal interface for antigen uptake during immersion exposure, making it the most direct indicator of early local immune activation [14].

The KEGG enrichment analysis identified DEGs across multiple immune- and signaling-associated pathways, along with their enrichment factors (EFs) (Table 1).

The KEGG pathway analysis identified several immune-related pathways that were significantly enriched in the bath intestine compared with controls (Table 1). The most prominent were *calcium signaling* (64 DEGs; EF = 1.41), *cell adhesion molecules (CAMs)* (49 DEGs; EF = 1.54), and *phagosome* (47 DEGs; EF = 1.27), indicating enhanced immune cell communication, epithelial barrier remodeling, and antigen processing following bath immunization. *Cytokine–cytokine receptor interaction* (38 DEGs; EF = 1.05) also showed slight enrichment, consistent with the modulation of cytokine-mediated signaling.

Several additional immune pathways contained DEGs but were not overrepresented relative to the pathway size (EF < 1). These included *MAPK signaling* (54 DEGs; EF = 0.96), *Toll-like receptor signaling* (12 DEGs; EF = 0.68), *NOD-like receptor signaling* (10 DEGs; EF = 0.37), *RIG-I-like receptor signaling* (5 DEGs; EF = 0.52), and *C-type lectin receptor signaling* (15 DEGs; EF = 0.67), indicating transcriptional activity across multiple innate recognition pathways. Broader cellular defense and stress response pathways such as *lysosome* (169 DEGs; EF = 0.59), *Salmonella infection* (25 DEGs; EF = 0.58), *apoptosis* (18 DEGs; EF = 0.64), *cellular senescence* (25 DEGs; EF = 0.77), and the *cytosolic DNA-sensing pathway* (2 DEGs; EF = 0.28) also showed DEG representation without enrichment. Similarly, the *intestinal immune network for IgA production* (13 DEGs; EF = 0.88) exhibited transcriptional modulation despite the absence of overrepresentation.

Overall, bath immunization induced the coordinated modulation of pathways associated with innate recognition, signaling, cell adhesion, antigen processing, and mucosal immune function. Enriched pathways (EF > 1) highlight key axes of overrepresented immune activity, whereas additional pathways with multiple DEGs but EF < 1 reflect the broader remodeling of intestinal immune and stress response networks.

Beyond this, Table 2 was produced to display differentially expressed immune-related genes identified from immune-associated KEGG pathways, including Ensembl gene IDs, gene descriptions, and fold-changes at 7 days post-vaccination, organized into eight predefined immune categories.

This pathway was prioritized because it uniquely captured the coordinated regulation of mucosal immunoglobulin-associated signaling, relevant cytokines, antigen presentation, and epithelial transport machinery, which were central to the intestinal immune responses elicited by bath immunization and were not as comprehensively represented in other enriched KEGG pathways.

The pathway illustrates conserved components of mucosal immune activation, including antigen uptake and presentation, cytokine-mediated signaling, lymphocyte activation, and epithelial immunoglobulin transport machinery. In channel catfish, these transcriptional signatures are most consistent with IgM-dominant mucosal immune responses rather than classical mammalian IgA biology. The differential upregulation of key components involved in T-cell activation (MHC class II), cytokine signaling (IL-6), receptor–ligand interactions (integrin α4β7, CXCR4–CXCL12), and epithelial IgM transport (pIgR) indicates the active modulation of mucosal adaptive immunity following bath immunization.

## 4. Discussion

*Edwardsiella piscicida* remains a major pathogen of cultured fish and an important challenge for catfish aquaculture, particularly because its intracellular lifestyle and species-level misidentification can complicate surveillance and control [15,16]. Within this context, the χ16016 platform is relevant not simply as another attenuated strain but as a regulated-lysis vaccine designed to combine transient in-host replication and antigen delivery with loss of viability after host entry, thereby improving biological containment relative to conventional live vaccines [16]. Previous work established the safety, biological containment, immunogenicity, and protective efficacy of χ16016 in channel catfish and, together with related *Edwardsiella* vaccine vector studies and the 2023 catfish study, demonstrated that properly attenuated *E. piscicida* strains elicit adaptive immune engagement, including systemic and mucosal IgM responses with measurable protection in challenge models, supporting a broader framework for interpreting the present transcriptomic findings [2,17].

The present study extends previous work on χ16016 by identifying early vaccination-associated transcriptional responses in the intestine and kidney at 7 days post-vaccination. These data should be interpreted as transcriptomic associations rather than direct evidence of protective mechanisms. The safety, immunogenicity, and challenge protection of χ16016 were established previously, whereas the present study was designed to define tissue- and route-associated host gene expression patterns after vaccination. Therefore, the pathways identified here may represent biologically plausible components of vaccine-induced immune remodeling, but they do not establish causal mechanisms of protection.

Intestinal tissue displayed stronger transcriptional responsiveness than kidney tissue, consistent with the central role of teleost mucosal surfaces in sensing environmental antigens and coordinating local and systemic immunity [18,19,20]. By contrast, kidney responses were more constrained, which is compatible with the kidney’s role as a major lympho-hematopoietic and systemic immune organ rather than a primary mucosal interface [20,21]. The broader transcriptional disruption observed after intracoelomic vaccination likely reflects a combination of systemic antigen exposure, a higher localized dose, and sedation-associated stress rather than the route alone when compared with bath immersion [17]. Accordingly, a larger DEG set after IC delivery likely reflects broader systemic exposure rather than inherently superior immunity.

Notably, KEGG pathway enrichment analyses were primarily performed in the intestine following bath immersion vaccination compared to controls, whereas other comparisons were evaluated using GO-based analyses. In the intestine following bath immersion vaccination, the transcriptomic patterns at 7 dpv were consistent with what might be expected during a transition from early innate activation toward antigen processing. The enrichment of the *phagosome*, *lysosome*, *cell adhesion*, *cytokine signaling*, and *MAPK-associated* pathways suggests transcriptional signatures of antigen uptake, intracellular processing, leukocyte positioning, and early adaptive priming. This interpretation is consistent with the expected progression of teleost vaccine responses, in which intense PRR-driven activation occurs early, followed by antigen presentation and tissue remodeling programs that are more detectable at later early time points [19,20,22]. The present transcriptomes identify pathway-level patterns that are consistent with immune activation.

In the bath immersion intestine comparison, the enrichment of the KEGG *intestinal immune network for IgA production* pathway must be interpreted within channel catfish immunobiology. Teleosts generally lack classical mammalian IgA, and, although IgT/IgZ functions as a major mucosal immunoglobulin in many fish, channel catfish appear to rely primarily on IgM and IgD and do not show a canonical IgT/IgZ locus [23,24,25]. Therefore, this pathway likely represents an orthology-based map of conserved mucosal immunoglobulin-associated machinery, with defenses potentially relying on IgM (and possibly specialized IgD biology) rather than mammalian-style IgA biology [25]. Our previous study with χ16016 demonstrated vaccine-induced increases in both serum and mucosal IgM, although whether the transcriptomic signatures observed here directly contribute to those responses requires functional validation [2].

This interpretation is strengthened by the present upregulation of pIgR, MHC class II-related components, IL-6, and other genes linked to mucosal antibody deployment. Because pIgR mediates the transepithelial transport of mucosal immunoglobulins in teleosts and IL-6 can support B-cell differentiation and IgM responses, these transcriptional changes are consistent with pathways involved in IgM-mediated mucosal responses [2,25,26,27].

The current data also support coordinated leukocyte recruitment and positioning within the intestinal tissue. The upregulation of adhesion- and trafficking-related genes, including integrin-associated molecules, VCAM1, ICAM2, PECAM1, and the CXCL12–CXCR4 axis, is mechanistically coherent with the establishment of mucosal immune organization after bath vaccination [18,20]. Although these transcriptomic patterns do not by themselves resolve cell types or tissue localization, they suggest that immersion exposure may be associated with structured mucosal recruitment programs rather than minimal responses.

Another important feature observed in the bath-immunized intestine is the convergence of complement, scavenger receptor, and intracellular signaling signatures with antigen processing pathways. This interpretation is further supported by the enrichment of antigen processing-related pathways and genes, including the *phagosome* and *lysosome* pathways, cathepsins, and both MHC class I and class II components. This suggests transcriptional associations with antigen uptake, processing, and presentation during the post-vaccination phase.

The increased expression of C3, scavenger-type recognition molecules, cathepsin-associated processing functions, and the *phagosome/lysosome* pathways is consistent with transcriptional patterns associated with opsonophagocytic clearance [28,29,30]. At the same time, the regulation of MAPK-associated components and CSF1-related signaling supports a model in which coordinated transcriptional responses such as phagocyte activation, survival, and inflammatory calibration occur together rather than as isolated events [31]. These patterns are consistent with coordinated antigen handling rather than uncontrolled inflammation. These pathway-level findings should be interpreted specifically in the context of the bath immersion intestinal response, as other tissues and vaccination regimens were primarily evaluated through GO-based analyses rather than KEGG enrichment profiles.

Furthermore, IC vaccination, which also involves sedation and direct intracoelomic antigen deposition at higher local concentrations, is associated with broader systemic transcriptional remodeling, whereas bath immersion induces a more focused intestinal transcriptional program involving genes related to antigen handling, chemokine signaling, adhesion pathways, pIgR-linked immunoglobulin transport, and complement activation. This distinction is especially relevant for aquaculture because immersion vaccination is operationally practical, and our data show that it elicits detectable transcriptional responses rather than minimal changes [2,32,33]. These transcriptomic patterns are consistent with mucosal immune pathways described in teleost fish, although functional validation would be needed to establish their biological significance and confirm safety for field deployment [2,33].

This study has several important limitations. The experimental design confounded the vaccination route with regimen variables including the antigen dose, exposure duration, handling intensity, and sedation use. These procedural differences are inherent to each delivery method as practiced in commercial aquaculture, making the removal of confounds impossible and precluding the attribution of transcriptional differences exclusively to the route of antigen entry. The broader transcriptional changes observed after intracoelomic vaccination should not be interpreted as superior immune activation but likely reflect systemic disruption from these inherent procedural differences rather than more effective immunity.

Additional limitations include the use of pooled fish samples with only three biological replicates per group, the absence of tank-level replication, single-timepoint sampling at 7 dpv, the lack of qPCR/functional validation, and the reliance on mammalian-based annotations, which may not accurately reflect teleost immune functions, particularly for mucosal immunity mechanisms. Growth performance was not quantitatively assessed in this study, which was designed to examine early tissue transcriptomic responses at 7 dpv. Although no obvious gross differences were observed between control and vaccinated fish during routine monitoring, future studies should include body weight gain, feed intake, and feed conversion to evaluate the production impacts of χ16016 immunization. Functional validation studies should include time-course experiments and challenge studies to assess whether transcriptomic patterns translate to measurable immune outcomes.

## 5. Conclusions

This study defines how a regulated-lysis *Edwardsiella piscicida* vaccine (χ16016) alters host gene expression in key immune tissues of channel catfish at an early post-vaccination stage (7 days post-immunization). The results show that the vaccination route influences where immune-relevant transcriptional responses are concentrated rather than simply increasing the overall magnitude of transcriptional change. Intracoelomic injection was associated with a larger number and wider dispersion of differentially expressed genes across tissues, whereas bath immersion produced more focused transcriptional changes in the intestine, including the differential expression of genes linked to antigen handling, complement components, cell adhesion, and immunoglobulin transport, reflecting the distinct immune engagement profiles of the two most operationally relevant delivery methods in commercial catfish aquaculture. However, the broader transcriptional changes following IC vaccination likely reflect systemic perturbation from higher antigen loads, sedation, and handling stress rather than superior immune activation.

These transcriptomic patterns represent associations rather than demonstrated protective mechanisms. Bath vaccination induced the consistent upregulation of genes involved in mucosal immunoglobulin handling, including polymeric immunoglobulin receptor (pIgR), MHC class II components, complement C3, and chemokine signaling—patterns that are compatible with IgM-based mucosal defense in channel catfish. These transcriptional signatures are biologically plausible for mucosal immune responses, although functional validation is required to establish their biological significance.

From a production standpoint, immersion vaccination is operationally attractive for large-scale catfish farming. These results show that bath delivery is associated with coordinated intestinal transcriptional responses rather than weak or nonspecific changes. However, direct evidence linking these specific transcriptional profiles to field-relevant protection, safety under production conditions, and cost–benefit performance is needed before these data can support deployment recommendations for edwardsiellosis control in commercial catfish aquaculture. Together, these data provide a transcriptomic context for the previously observed protection and establish a foundation for future functional validation studies aimed at understanding vaccine-induced immune mechanisms in teleost species.

## Figures and Tables

**Table 1:** Counts of DEGs across the various immune-related KEGG pathways in Bath vs. Control Intestine. Immune-related KEGG pathways identified in the bath-immunized intestine compared to the control are shown with the KEGG pathway ID, total number of significant DEGs, and enrichment factor. The enrichment factor reflects the ratio of observed to expected DEGs within each pathway. Pathways with enrichment factors > 1 indicate the overrepresentation of DEGs, whereas values < 1 indicate relative underrepresentation.

Pathway	Pathway ID	Total DEGs	Enrichment Factor
*Calcium signaling*	ipu04020	64	1.41
*Cell adhesion molecules (CAMs)*	ipu04514	49	1.54
*Phagosome*	pipu04145	47	1.27
*Cytokine–cytokine receptor signaling*	ipu04060	38	1.05
*MAPK signaling*	ipu04010	54	0.96
*Toll-like receptor signaling*	ipu04620	12	0.68
*NOD-like receptor signaling*	ipu04621	10	0.37
*RIG-I-like receptor signaling pathway*	ipu04622	5	0.52
*C-type lectin receptor signaling pathway*	ipu04625	15	0.67
*Lysosome*	ipu04142	169	0.59
*Salmonella infection pathway*	ipu05132	25	0.58
*Intestinal immune network for IgA production*	ipu04672	13	0.88
*Cytosolic DNA-sensing pathway*	ipu04623	2	0.28
*Apoptosis*	ipu04210	18	0.64
*Cellular senescence*	ipu04218	25	0.77

**Table 2:** Differentially expressed immune-related genes in Bath vs. Control intestine at 7 dpv. The table lists significant immune-related DEGs identified from KEGG pathways, organized by Ensembl Gene ID, gene name, full name with function, and fold change (FC) at 7 days post-vaccination (dpv). Genes are grouped into eight predefined immune categories: pattern recognition receptors (PRRs), cytokines/chemokines, cytokine/chemokine receptors, signal transduction molecules, immune-related transcription factors, complements, antigen processing and presentation, and effector/antimicrobial proteins.

Ensembl Gene ID	Gene Name	Full Name + Function	FC (7 dpv)
**Pattern Recognition Receptors (PRRs)**			
ENSIPUG00000015277	MR	Mannose receptor C-type lectin—PRR that binds mannose on microbes and apoptotic cells	3.65
ENSIPUG00000024330	CD36	Cluster of differentiation 36—Scavenger receptor for oxidized lipids and apoptotic cells	3.61
ENSIPUG00000023552	Collectins	Collectin family—Soluble C-type lectin involved in innate immunity	10.63
**Cytokines and Chemokines**			
ENSIPUG00000004332	IL-6	Interleukin-6—Pro-inflammatory cytokine	6.11
ENSIPUG00000000241	CXCL12	C-X-C motif chemokine 12—Chemokine that attracts hematopoietic cells	1.88
ENSIPUG00000022525	TGFβ	Transforming growth factor beta—Cytokine that modulates immune responses	−2.21
ENSIPUG00000020436	CCL19	C-C motif chemokine 19—Chemokine that guides T-cell migration	−1.28
ENSIPUG00000003184	CCL3	C-C motif chemokine 3—Chemokine that recruits neutrophils	−7.34
novel.1741	CCL20	C-C motif chemokine 20—Chemokine recruiting dendritic cells, T cells, and neutrophils	2.40
ENSIPUG00000012017	IL17F	Interleukin-17F—Pro-inflammatory cytokine	−2.88
ENSIPUG00000024495	CSF1	Colony-stimulating factor 1—Macrophage-stimulating growth factor	1.74
ENSIPUG00000020916	APRIL	A proliferation-inducing ligand—B-cell growth and differentiation factor	−2.09
ENSIPUG00000022162	TRAILR (DR4/5)	TNF-related apoptosis-inducing ligand receptor	1.35
**Cytokine/Chemokine Receptors**			
ENSIPUG00000010000	CXCR4	C-X-C motif chemokine receptor 4—Receptor for CXCL12	1.66
ENSIPUG00000004362	IL6R	Interleukin-6 receptor—Receptor for IL-6 signaling	1.70
ENSIPUG00000000617	CCR4	C-C chemokine receptor type 4—Mediates Th2-cell trafficking	−2.09
ENSIPUG00000024940	CCR5	C-C chemokine receptor type 5—Guides immune cells to inflammation	−6.56
**Signal Transduction Molecules**			
ENSIPUG00000015786	JNK (MAPK8/9)	c-Jun N-terminal kinase (JNK)—Stress-activated MAP kinase	7.63
ENSIPUG00000006710	p38 (MAPK14)	p38 MAP kinase 14—MAP kinase involved in inflammation	−1.27
ENSIPUG00000003405	PI3K	Phosphatidylinositol-3-kinase—Key signaling lipid kinase	2.69
ENSIPUG00000013954	Ras	Ras—Small GTPase that regulates proliferation	1.33
ENSIPUG00000013925	RasGRF	Ras guanyl-releasing factor—GTP-exchange factor that activates Ras	2.34
ENSIPUG00000015033	CALM (CaM)	Calmodulin—Calcium-binding protein	5.12
ENSIPUG00000006002	PAK1	p21-activated kinase 1—Serine/threonine kinase downstream of Rac/Cdc42	1.71
ENSIPUG00000018621	CARD9	Caspase recruitment domain-containing protein 9—Adaptor protein in innate immune signaling	−1.54
ENSIPUG00000005687	ITGA4	Integrin alpha 4—Integrin subunit that mediates leukocyte adhesion (forms α4β7 heterodimer)	2.18
ENSIPUG00000012647	cPLA2	Cytosolic phospholipase A2—Releases arachidonic acid	−5.04
ENSIPUG00000015287	PKC	Protein kinase C family—Serine/threonine protein kinase family	1.74
ENSIPUG00000009525	PECAM1	Platelet endothelial cell adhesion molecule 1—Mediates leukocyte transmigration across endothelium	1.83
ENSIPUG00000012628	ICAM2	Intercellular adhesion molecule 2—Mediates leukocyte adhesion to endothelial cells	3.63
ENSIPUG00000024811	VCAM1	Vascular cell adhesion molecule 1—Promotes leukocyte recruitment	2.18
ENSIPUG00000023961	SELP	P-selectin—Mediates leukocyte rolling	2.30
ENSIPUG00000007024	ESAM	Endothelial cell-selective adhesion molecule—Mediates leukocyte transmigration	2.43
ENSIPUG00000022530	STIM	Stromal interaction molecule—ER calcium sensor that triggers store-operated Ca^2+^ entry	−2.33
ENSIPUG00000008458	TRPML	Transient receptor potential mucolipin—Lysosomal mucolipin channel	1.66
**Immune-Related Transcriptional Regulators**			
ENSIPUG00000012538	IκBα (NFKBIA)	Inhibitor of NF-κB alpha—Transcription factor inhibitor	1.27
ENSIPUG00000016082	AP1 (cFos)	c-Fos—c-Jun/c-Fos transcription factor complex	−1.30
ENSIPUG00000015251	NFAT	Nuclear factor activated T cells (NFAT)—Nuclear factor of activated T cells	1.50
ENSIPUG00000002771	Nur77	NR4A1 (Nur77)—Orphan nuclear receptor involved in T-cell signaling	1.66
ENSIPUG00000013747	MEF2C	Myocyte enhancer factor 2C—Transcription factor regulating immune-related genes	−1.18
ENSIPUG00000014722	LITAF	Lipid-responsive transcription factor—Involved in inflammation	1.24
**Complement System**			
ENSIPUG00000009619	C3	Complement component 3—Central complement protein cleaved into fragments that opsonize targets	10.34
**Antigen Processing and Presentation**			
ENSIPUG00000023147	MHCI	Major histocompatibility complex class I—MHC I antigen-presenting complex	1.38
ENSIPUG00000004387			1.30
ENSIPUG00000022867			1.28
ENSIPUG00000004298			1.64
ENSIPUG00000021534	MHCII	Major histocompatibility complex class II—MHC II antigen-presenting complex	2.55
ENSIPUG00000021529			1.73
ENSIPUG00000010998	cathepsin	Cathepsin—Lysosomal protease for antigen processing	3.24
**Effector Molecules/Antimicrobial Proteins**			
novel.1048	pIgR	Polymeric immunoglobulin receptor—Receptor that transports IgA/IgM	2.79

## Data Availability

The original contributions presented in this study are included in the article/Supplementary Material. Further inquiries can be directed to the corresponding author.

## Supplementary Materials

The following supporting information can be downloaded at https://www.mdpi.com/article/10.3390/vaccines14050410/s1. Figure S1: Gene Ontology (GO) enrichment analysis in intestinal and kidney tissues following bath and intracoelomic immunization; Figure S2: GO enrichment analysis of Bath vs. IC-immunized kidney.
