# Supplementary material for: Route-Dependent Mucosal and Systemic Immune Remodeling Induced by a Regulated-Lysis Edwardsiella piscicida Vaccine in Channel Catfish
Source: Vaccines (Basel). 2026 May 1;14(5):410. doi: 10.3390/vaccines14050410 (PMC13211737; doi:10.3390/vaccines14050410)
Supplement: Supplementary file 1 [file vaccines-14-00410-s001.zip › vaccines-4262316-supplementary.pdf]

Supplementary Information

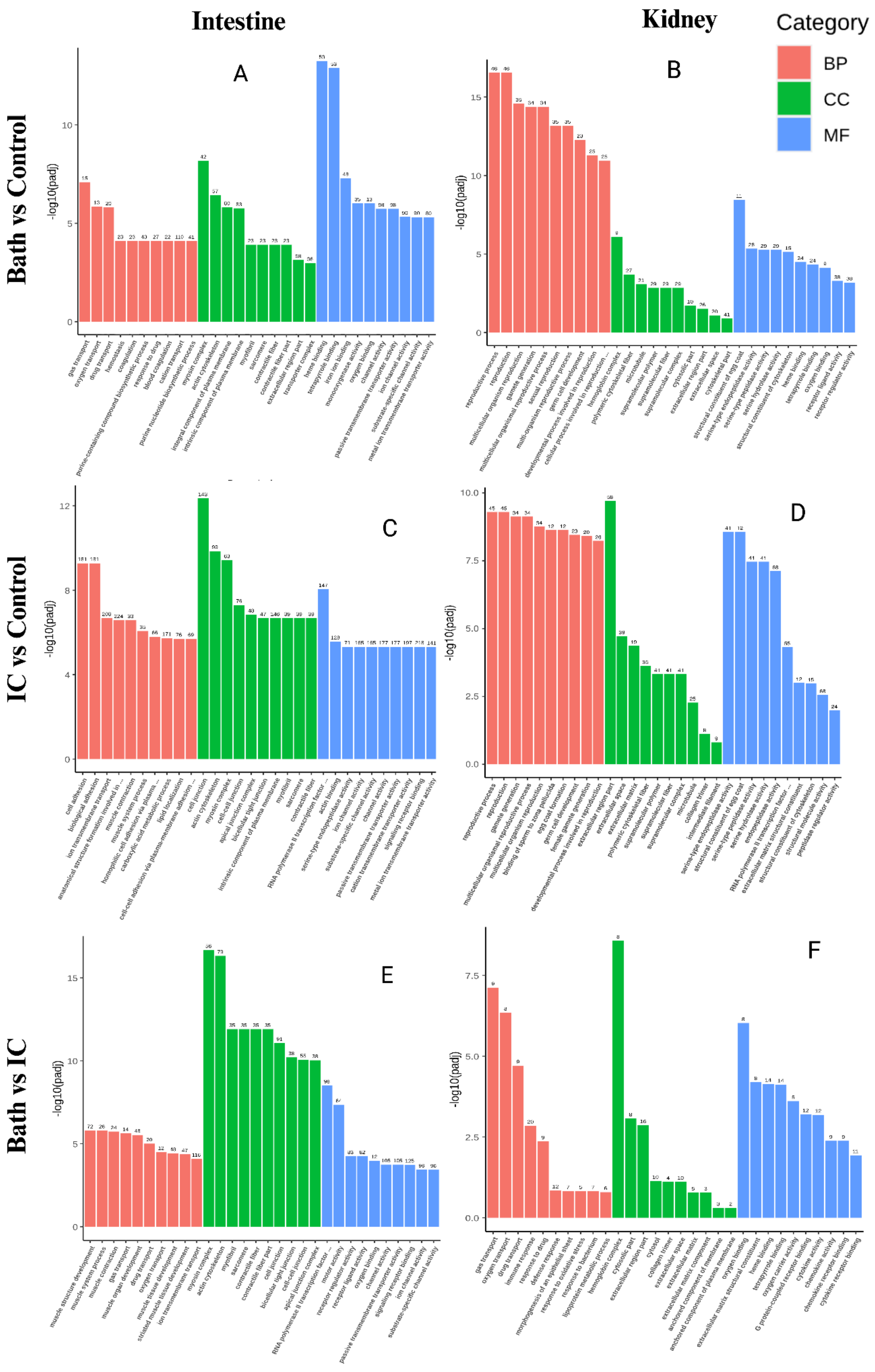

Supplementary Figure S1. Gene Ontology (GO) enrichment analysis in intestinal and kidney tissues following bath and intracoelomic immunization.

Bar plots (A–F) display the top 10 significantly enriched Gene Ontology (GO) terms within each functional category—Biological Process (BP), Cellular Component (CC), and Molecular Function (MF)—for all six experimental comparisons. Panels A, C, and E represent intestinal tissue (A: Bath vs Control; C: IC vs Control; E: Bath vs IC), while panels B, D, and F represent kidney tissue (B: Bath vs Control; D: IC vs Control; F: Bath vs IC). Bars are colored according to GO category: BP (red), CC (green), and MF (blue). The x-axis lists enriched GO terms, and the y-axis represents enrichment significance ($-\log_{10}$ adjusted p-value). Numbers above each bar indicate the number of differentially expressed genes (DEGs) associated with each term.

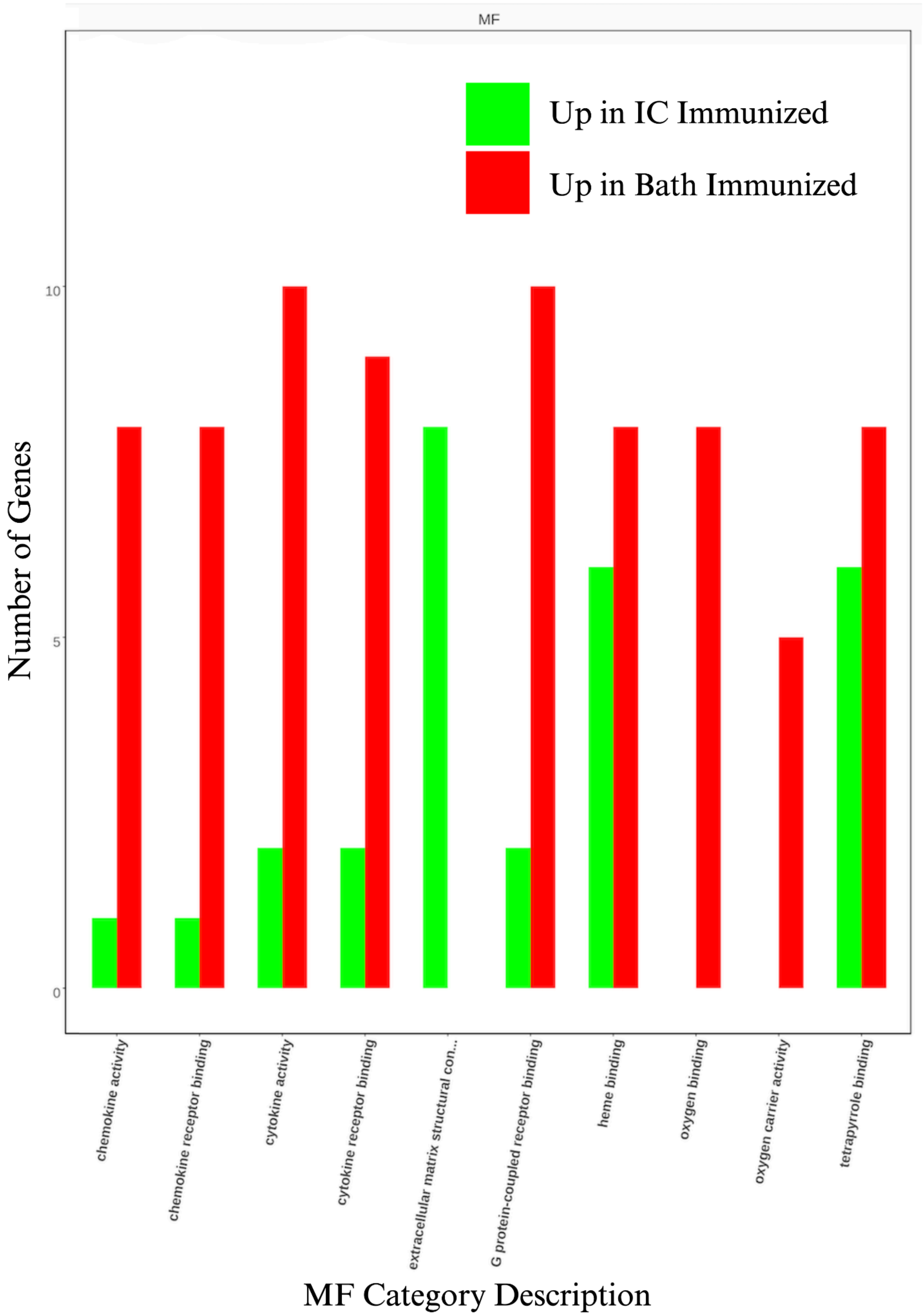

Supplemental Figure S2: GO enrichment analysis of Bath vs IC immunized kidney. Bar graph showing the number of differentially expressed genes within the Molecular Function (MF) category. Red bars indicate genes upregulated in bath immunized kidney, and green bars indicate genes upregulated in IC immunized kidney.
